# The Effect of Continuous Versus Periodic Vital Sign Monitoring on Disease Severity of Patients with an Unplanned ICU Transfer

**DOI:** 10.1007/s10916-023-01934-3

**Published:** 2023-03-31

**Authors:** Yassin Eddahchouri, Roel V. Peelen, Mats Koeneman, Alec van Veenendaal, Harry van Goor, Sebastian J. H. Bredie, Hugo Touw

**Affiliations:** 1 Department of Surgery, Radboud university medical center, PO Box 9101, 618, Nijmegen, 6500 HB The Netherlands; 2 Department of Internal Medicine, Radboud university medical center, Nijmegen, The Netherlands; 3 Department of Intensive Care, Radboud university medical center, Nijmegen, The Netherlands

**Keywords:** General ward, Continuous wireless monitoring, Vital signs, Wearables, ICU, Rapid Response team

## Abstract

Continuous vital sign monitoring (CM) may detect ward patient’s deterioration earlier than periodic monitoring. This could result in timely ICU transfers or in a transfer delay due to misperceived higher level of care on the ward. The primary objective of this study was to compare patient’s disease severity upon unplanned ICU transfer, before and after CM implementation. We included a one-year period before and after CM implementation between August 1, 2017 – July 31, 2019. Before implementation, surgical and internal medicine patients’ vital signs were periodically monitored, compared to continuous monitoring with wireless linkage to hospital systems after implementation. In both periods the same early warning score (EWS) protocol was in place. Primary outcome was disease severity scores upon ICU transfer. Secondary outcomes were ICU and hospital length of stay, incidence of mechanical ventilation and ICU mortality. In the two one-year periods 93 and 59 unplanned ICU transfer episodes were included, respectively. Median SOFA (3 (2–6) vs 4 (2–7), p = .574), APACHE II (17 (14–20) vs 16 (14–21), p = .824) and APACHE IV (59 (46–67) vs 50 (36–65), p = .187) were comparable between both periods, as were the median ICU LOS (3.0 (1.7–5.8) vs 3.1 (1.6–6.1), p.962), hospital LOS (23.6 (11.5–38.0) vs 19 (13.9–39.2), p = .880), incidence of mechanical ventilation (28 (47%) vs 22 (54%), p.490), and ICU mortality (11 (13%) vs 10 (19%), p.420). This study shows no difference in disease severity upon unplanned ICU transfer after CM implementation for patients who have deteriorated on the ward.

**Supplementary Information:**

The online version contains supplementary material available at 10.1007/s10916-023-01934-3.

## Introduction

Vital sign deterioration often precedes adverse events on hospital wards and its early detection provides an opportunity to intervene, potentially preventing these events [1–5]. Hospitals have adopted periodic vital sign monitoring and early warning scores (EWS), including Rapid Response Team (RRT) deployment, to improve clinical outcome. The exact added value of these systems to patients’ outcomes remains uncertain [6, 7], presumably due to poor protocol compliance, inaccurate recordings and the periodic nature of the vital sign measurements [8–11], which can result in clinical deterioration to progress unnoticed [12–14]. Continuous vital sign monitoring (CM) on general wards has been advocated to enable early detection of clinical deterioration and to improve patient outcome [15–18]. In 2010 we introduced a Modified Early Warning Score (MEWS) in our hospital which gradually evolved based on existing EWSs and local expert opinion [19–21]. Its final version was implemented in 2014 (Appendix 1). In 2018 continuous monitoring of respiration rate, SpO2, heart rate and blood pressure were introduced on a surgical and internal medicine ward. CM became the basis for the MEWS calculations. Like in previous studies, we recently showed in a before-after study that the implementation of CM on the hospital ward was associated with a significant reduction of unplanned Intensive Care Unit (ICU) transfers (93 vs 59 unique transfers and 84 (3.4%) vs 54 (2.3%) hospital admissions with a transfer (p = 0.03) [19, 22, 23]. CM could indeed have a positive effect in supporting physicians and nurses to be more vigilant and result in earlier detection of clinical deterioration. Its positive effect could prompt interventions, such as timely ICU transfer, resulting in lower disease severity upon ICU arrival.

Opposingly, CM could have a negative effect. Continuous vital sign monitoring could unintentionally prolong the management of deteriorating patients on the ward due to a misperceived higher level of care. CM could cause delayed ICU transfer resulting in higher disease severity upon ICU arrival. The latter has been a major concern of the nursing staff on the ward during implementation of continuous monitoring. The nursing staff feels, and rightfully so, that they are not equipped to care for critically ill patients [24].

Disease severity scores correlate with ICU mortality [25–28]. Delayed ICU transfer has been associated with increased disease severity scores [29] and increased ICU mortality in critically ill patients [30–32]. Whether CM compared to periodic monitoring leads to timely or delayed ICU transfers may be reflected by decreased or increased disease severity scores. Studies that report on the effects of implementation of CM on disease severity at the moment of unplanned ICU transfers are scarce.

In this study we primarily aim to assess the effect of CM on disease severity of patients at the time of unplanned ICU transfer. We hypothesize that disease severity upon ICU transfer, reflected by SOFA and APACHE, does not increase after CM introduction. Second, we aim to assess the effect of CM on the ICU and hospital length of stay, the need for mechanical ventilation and ICU mortality.

## Methods

### Study design

We performed a before-and-after study in a large tertiary referral hospital in the Netherlands. We included patients who underwent an unplanned transfer from the surgical or internal medicine ward (34 and 26 beds, respectively) to the ICU or Medium Care Unit (MCU). On the surgical ward mainly patients with upper gastrointestinal- and/or hepato-pancreatic-biliary cancer were admitted. General internal medicine, rheumatology, and infectious disease patients were admitted to the internal medicine ward.

Electronic medical record (EMR) notes of the ward and ICU clinicians were reviewed. An unplanned ICU (uICU) transfer was defined as an unforeseen transfer to the ICU or MCU due to clinical deterioration of the patient or after (re)operation for a complication. Ultimately we only included patients who were admitted to the ICU or MCU for more than eight hours to make sure there was enough time to acquire the parameters needed for the calculation of the disease severity scores. The main difference between the ICU and MCU is that the MCU has no mechanical ventilation resources.

We included a one-year period before CM implementation (pre), August 1, 2017 – July 31, 2018, and a one-year period after CM implementation (post), August 1, 2018 – July 31, 2019. The study has been approved by the local ethics committee of the Radboudumc (case number: 2018–4330).

### Vital sign measurements and processing

Before CM implementation, nurses manually measured and registered the vital sign parameters in the EMR (Hyperspace, Epic systems corporation, Verona, Wisconsin, USA), using a blood pressure measuring device with a pulse oximeter (heart rate (HR), blood pressure (BP) and oxygen saturation (SpO2)) (Dinamap V100, GE healthcare, Chicago, Illinois, USA or CONTEC 08A, CONTEC Medical Systems Co., Ltd, Qinhuangdao, Hebei, China), an ear thermometer (core temperature (T)) (Covidien Genius 2, Covidien, Dublin, Ireland), and by visual inspection (respiration rate (RR)). In addition to HR, BP, SpO2, RR and T, the supplemental oxygen delivery (L/min) as well as the AVPU score (Alert, Delirious, Voice, Pain or Unresponsive) were collected to acquire a MEWS (Appendix 1). The MEWS is automatically calculated if at least HR, BP, SpO2, RR and T were registered and validated at one timepoint using zero scores for a missing AVPU score and/or missing supplemental oxygen.

We used the same MEWS protocol during both years. This stipulates a vital sign recording and MEWS calculation every 8 h in all patients at baseline. Elevated MEWS (3–5) requires a measurement every 4 h, while hourly measurements and consulting the ward physician or calling the rapid response team (RRT, (intensive care physician and critical care registered nurse)) are mandatory after alarming MEWS (≥ 6).

After CM implementation in august 2018, patients were monitored with a wearable device (VisiMobile, Sotera Wireless, San Diego, CA, USA). This wrist device continuously measures HR, BP, SpO2 and RR. Once every minute, all vital signs were automatically sent to the EMR and were available for periodic validation by the nurses. The MEWS is automatically calculated if HR, BP, SpO2, RR and a manually measured core temperature were registered and validated at one timepoint. Therefore, the used EMR database contained two types of validated vital sign sets 1) sets containing all mandatory vital signs with an automatically calculated MEWS and 2) extra sets without all vital signs that are mandatory for automatic MEWS calculation, resulting in vital sign sets without a MEWS calculation.

Monitors at the nurses’ stations provided clinicians around the clock real-time vital sign data (Appendix 2). The vital sign measurements and trends of the preceding 96 h were also available. Single channel alarms on the monitors or the wrist device were generated when a vital parameter exceeded a preset range (Appendix 3). Patient exclusion or disconnection of the device were only done after clinical assessment by a nurse, or a physician involved in the study. Predefined reasons to disengage CM were a hyperactive delirium during transfer, skin problems and patient’s refusal to be continuously monitored. Patients who underwent elective surgery were connected to the wearable device after surgery.

### Data collection for disease severity scores

Data was collected (retrospectively) from the Dutch National Intensive Care Evaluation (NICE) Registry. NICE registry collects data concerning disease severity and outcomes of admissions to the adult ICUs of all Dutch hospitals, with the aim to monitor and improve care. Data of each individual patient was (prospectively) registered by the attending physician. MCU patients are not included in the NICE registry. Data of these patients was retrospectively obtained by EMR review (YE, RP) using the same format as the national registry. The datasets included demographics, transfer diagnoses, comorbidities, length of hospital and ICU stay, vital signs at the time of ICU transfer, ICU interventions, ICU mortality and disease severity scores (SOFA [33], APACHE II [25, 34], and APACHE IV [35]) at the time of ICU transfer.

### Study outcome parameters

Primary outcomes were the SOFA, APACHE II and APACHE IV at the moment of the ICU transfer. Secondary outcomes were the length of hospital and ICU stay, the incidence of mechanical ventilation and ICU mortality. Other outcomes were the frequency (times/day) and quality of the validated vital sign sets and time between first alarming MEWS and ICU transfer in the 24 h preceding the ICU admission.

### Statistical analysis

Data are presented as mean [SD], median [IQR] or n (%). The Mann–Whitney U test was used to compare non-normally distributed data, the Student’s t-test was used for normally distributed data and the Chi-square was used for categorial variables. A p-value below 0.05 was considered statistically significant. All analyses were performed using statistical package for the social sciences (SPSS) package version 25.0 (SPSS, Inc, Chicago, IL).

## Results

### Patient characteristics

We identified 136 patients with an unplanned ICU transfer. In the pre period 82 patients underwent a total of 93 uICU transfers and 84 hospital admissions. In the post period 54 patients underwent a total of 59 uICU transfers and 54 hospital admissions. No differences in patient characteristics were found between the groups (Table 1).

**Table 1:** Patient characteristics pre and post implementation of CM

	**Total**	**pre**	**post**	***p*-value**
Unique patients, n	136	82	54	
Hospital admissions, n	138	84	54	
Unplanned ICU transfers, n	152	93	59	
Age, median years (IQR)	67 (58 – 72)	67 (58 – 74)	64 (55 – 71)	.182
Female gender, n (%)	65 (43)	38 (41)	27 (46)	.552
Referring ward, n (%)				
− Surgical − Internal medicine	87 (57) 65 (43)	52 (56) 41 (44)	35 (59) 24 (41)	.679
Transfer ward				
− ICU − MCU	101 51	60 (65) 33 (35)	41 (69) 18 (31)	.527
GCS on ICU transfer, median (IQR)	15 (15 – 15)	15 (15 – 15)	15 (15 – 15)	.637
Mean vital signs on ICU transfer (SD)				
− Systolic BP − Heart rate	126 (32) 103 (23)	128 (31) 104 (22)	124 (34) 102 (25)	.619 .438
Diagnosis on ICU arrival				
− Pulmonary • Insufficiency unspecified • Embolism	57 56 1	34 34 0	23 22 1	.763
− Infectious • Sepsis • Pneumonia • Not specified	53 25 7 21	33 16 4 13	20 9 3 8	.842
− Bleeding	14	8	6	.745
− Cardio/vascular • AF • Heart failure • IHCA • Epidural induced hypotension	10 4 2 2 2	7 4 2 0 1	3 0 0 2 1	.554
− Depressed LOC	9	8	1	.79
− Other • Auto-inflammatory • (Auto-)intoxication • Bowel ischaemia/obstruction • Acute pain	9 3 2 3 1	3 1 1 1 0	6 2 1 2 1	.077

*ICU* intensive care unit, *IQR* interquartile range, *MCU* medium care unit, *GSC* Glasgow Coma Scale, *SD* standard deviation, *BP* blood pressure, *AF* atrial fibrillation, *IHCA* in hospital cardiac arrest, *LOC* level of consciousness

### Disease severity assessment

91 pre and 56 post uICU transfers were included in the disease severity analysis. Median SOFA was 3 (2–6) vs 4 (2–7), p = 0.574, APACHE II was 17 (14–20) vs 16 (14–21), p = 0.824 and APACHE IV was 59 (46–67) vs 50 (36–65), p = 0.187. There were no significant differences. The corresponding probability scores of ICU mortality are listed in Table 2.

**Table 2:** Disease severity score pre and post implementation of CM

**Disease severity scores**	**pre (n = 91)**		**Post (n = 56)**		
	**Median**	**IQR**	**Median**	**IQR**	***P-value***
SOFA	3	2 – 6	4	2 – 7	*.574*
APACHE II	17	14 – 20	16	14 – 21	*.824*
APACHE II probability*	.26	.13 – .38	.28	.17 – .40	*.474*
APACHE IV	59	46 – 67	50	36 – 65	*.187*
APACHE IV Probability*	.26	.13 – .44	.32	.14 – .45	*.446*

*SOFA* Sequential Organ Failure Assessment, *APACHE* Acute Physiology and Chronic Health Evaluation, *IQR* interquartile range

*ICU mortality probability

### Secondary outcomes

Median ICU LOS was 3.0 (1.7–5.8) vs 3.1 (1.6–6.1) days, p = 0.962, hospital LOS was 23.6 (11.5–38.0) vs 19.0 (13.9–39.2) days, p = 0.880, the incidence of mechanical ventilation was 28 (47%) vs 22 (54%), p.490, and the ICU mortality incidence was 11 (13%) vs 10 (19%), p = 0.420) compared between the pre and post patient population, respectively (Table 3).

**Table 3:** Length of hospital or ICU stay, ICU interventions and mortality in patients pre and post implementation of CM

	**n** **pre/post**	**pre**		**post**		
		**Median**	**IQR**	**Median**	**IQR**	***P-value***
ICU LOS, days	93/59*	3.0	1.7 – 5.8	3.1	1.6 – 6.1	*.962*
Hospital LOS, days	84/54**	23.6	11.5 – 38.0	19.0	13.9 – 39.2	*.880*
ICU Mortality (%)	82/54***	11 (13)		10 (19)		*.420*
Mechanical ventilation (%)	60/41****	28 (47)		22 (54)		*.490*

*ICU* intensive care unit, *LOS*: length of stay

*number of individual ICU transfers

**number of individual hospital admissions

***number of individual patients

****MCU (medium care unit) transfers are excluded, since there are no mechanical ventilation resources at the MCU

Time between the first alarming MEWS and the uICU transfer itself were comparable between both periods (8.8 h (2.6–18.9) vs 11.9 h (4.5–20.3), p = 0.185). During the 24 h before ICU admissions the nurses’ validation frequency of extra vital sign sets increased after CM implementation (Table 4). Moreover, after implementation of CM these validated extra vital sign sets without a MEWS calculation contained more vital signs than before implementation (systolic blood pressure (51% vs 77%), heart rate (60% vs 79%), respiratory rate (43% vs 75%), oxygen support (49% vs 79%) (Table 4). Comparable patterns were seen during night shifts (Table 4).

**Table 4:** Recording frequency of individual vital signs in the MEWS sets and in the extra sets in between, within 24 h of the adverse events

**All shifts**						
	Validated MEWSs^*^ pre	Validated MEWSs^*^ post	**20%** extra sets in between ^**^ pre	**39%** extra sets in between ^**^ post	**Total** vital sign sets*** pre	**Total** vital sign sets*** post
Number of uICUs						
Sets per uICU Mean Median	92 6.8 (3.3) 6 (5–9)	59 6.4 (3.5) 6 (4–8)	63 2.5 (2.0) 2 (1–3)	39 6.2 (8.4) 3 (2–6)	92 8.6 (4.3) 8 (5–11.5)	59 10.5 (8.2) 8 (6–13)
p-value	.429		.019		.536	
Systolic blood pressure, % (n)	100% (629)	100% (378)	51% (81)	77% (186)	90% (710)	91% (561)
Heart rate, % (n)	100% (629)	100% (378)	45% (72)	76% (182)	89% (701)	90% (560)
Oxygen saturation, % (n)	100% (629)	100% (378)	60% (96)	79% (190)	92% (725)	92% (568)
Temperature, % (n)	100% (629)	100% (378)	18% (28)	15% (37)	83% (657)	67% (415)
Respiratory rate, % (n)	100% (629)	100% (378)	43% (68)	75% (181)	88% (697)	90% (559)
AVPU performance, % (n)	100% (629)	100% (378)	18% (28)	17% (41)	83% (657)	68% (419)
Oxygen support, % (n)	99% (621)	99% (373)	49% (79)	79% (190)	89% (700)	84% (519)
MEWS calculated	100% (629)	100% (378)	-	-	80% (629)	61% (378)
**Night shifts**						
Number of uICUs						
Sets per uICU Mean Median	70 2.6 (1.7) 2 (1–3)	40 2.6 (1.7) 2 (1–3)	34 1.7 (1.1) 1 (1–2)	21 4.0 (4.8) 2 (2–4)	92 2.6 (2.4) 2 (1–4)	59 3.2 (4.1) 2 (0–5)
* p*-value	.865		.004		.746	
Systolic blood pressure, % (n)	100% (182)	100% (105)	41% (24)	86% (73)	85% (206)	93% (177)
Heart rate, % (n)	100% (182)	100% (105)	37% (22)	85% (72)	85% (204)	93% (177)
Oxygen saturation, % (n)	100% (182)	100% (105)	58% (34)	82% (70)	90% (216)	92% (175)
Temperature, % (n)	100% (182)	100% (105)	17% (10)	14% (12)	80% (192)	62% (117)
Respiratory rate, % (n)	100% (182)	100% (105)	46% (27)	82% (70)	87% (209)	92% (175)
AVPU performance, % (n)	100% (182)	100% (105)	22% (13)	13% (11)	81% (195)	61% (116)
Oxygen support, % (n)	99% (180)	99% (103)	61% (36)	62% (53)	90% (216)	82% (156)
MEWS calculated	100% (182)	100% (105)	-	-	76% (182)	55% (105)

^*^Only complete sets that did include all mandatory vital signs to calculate a MEWS

^**^Only the 20% and 39% (pre and post, respectively) of sets that were found to be incomplete and therefore did not include all mandatory vital signs to calculate a MEWS

***All vital sign recordings (complete (all mandatory vital signs present that are needed to calculate a MEWS) and incomplete sets (not all mandatory vital signs present to calculate a MEWS)). AVPU and oxygen support are not mandatory to calculate a MEWS

## Discussion

Continuous vital sign monitoring did not affect the disease severity score of clinically deteriorating patients on a surgical or internal medicine ward at the time of unplanned ICU transfer. CM also had no impact on ICU and hospital length of stay, incidence of mechanical ventilation or ICU mortality. Nurses validated more extra vital signs sets in the EMR and these extra vital sign sets contained also more vital sign parameters after CM implementation. While the nurses’ workflow has been impacted after implementation of CM; our results may suggest that continuous vital sign monitoring neither leads to preemptive nor delayed ICU transfer in clinically deteriorating patients.

Literature pertaining to the impact of CM on decision-making on hospital wards is scarce. Brown et al. reported that implementation of continuous respiratory and heart rate monitoring on a medical-surgical unit was associated with a significant decrease in total length of stay in the hospital and in intensive care unit days for transferred patients, as well as lower code blue rates [36]. They did not observe significant difference in unplanned ICU transfer or in disease severity score upon unplanned ICU arrival. Subbe et al. published a before-and-after study concerning continuous vital sign monitoring in combination with an electronic automated advisory and notification system. They found an association with a reduction in APACHE II scores at the time of ICU transfer [37]. Subbe et al. implied that deployment of the electronic automated advisory and notification system has the ability to significantly increase and expedite rapid response team deployment, which helped to detect clinical deterioration in an earlier phase.

Equivocal results in previous studies focusing on ICU transfer delay and patient outcome are potentially due to differences in study design, setting and definitions of delay. A slow ICU transfer, defined as a transfer 4 h or more after meeting the first physiologic threshold criteria on the ward, was associated with an increased APACHE II score, mortality and costs [29]. Others report an increase in mortality associated with delayed ICU transfer without higher APACHE II and SOFA scores upon ICU transfer [30, 38]. However these studies included patients who clinically deteriorated and were accepted for ICU transfer but stayed on the ward due to shortage in intensive care capacity. These patients were already identified as patients at risk. They probably received maximum treatment on the hospital ward with ICU team involvement, potentially improving physiological parameters and resulting in lower APACHE scores.

In concordance with our findings, previous data from our group also showed that vital sign parameters were frequently lacking in the extra validated vital sign sets [11]. Furthermore, the increased frequency of extra vital sign set validation after CM implementation was also showed by our research group in all patients with CM at the ward [23]. The clinical relevance of the increased validation frequency and vital sign presence of extra vital sign sets after CM implementation has yet to be determined.

In the previous study, we reported a 30 percent reduction in unplanned ICU transfers (93 vs 59 unique transfers and 84 (3.4%) vs 54 (2.3%) hospital admissions with a transfer (p = 0.03) [23]. Therefore, we hypothesize that the availability of continuous monitoring enabled physicians to treat more patients on the hospital ward. Our current study shows no difference in disease severity score upon ICU transfer. Combining our current and previous findings indicate that CM on the ward was successful and obviated the need for ICU transfer of patients, without delaying the transfer of others that eventually needed ICU care.

It is of paramount importance to realize that CM alone is not enough to eliminate ICU transfer delay or expedite the transfers of general ward patients. CM is just one facet of the safety system. Timely ICU transfer will probably require adjustments in RRT protocols, e.g., lower EWS thresholds for calling intensive care staff, as well as revision of ICU transfer and discharge criteria. Future studies are necessary to, for example, evaluate CM in combination with different predefined ICU transfer criteria on patient outcome. Moreover, ICU organization also impacts patient outcome. A closed format ICU staffed with dedicated critical care physicians is associated with lower hospital and ICU mortality and length of stay [39, 40]. We believe that the full potential of any safety system can only be realized when all the participating medical professionals are fully engaged and trained. General guidelines and protocols should always be tailored to the local situation.

### Strengths and limitations

Our study has several strengths and limitations. The study was conducted on two different hospital wards with a different patient mix increasing generalizability of the results. However, the single center study design may impede transferability to other hospitals. Another limitation was the retrospective before-and-after study design, which is vulnerable to bias [41]. Therefore a detailed analysis of all the factors that may have affected patient outcome was not possible. To overcome these limitations a multi-center RCT would be necessary to confirm the clinical effectiveness of CM and its effects on disease severity upon ICU transfer. Furthermore, we were not able to analyze the effect of CM on separate parameters of the disease severity scores. For example, Goldhill showed in a retrospective study that in the 24 h prior to ICU transfer only the respiratory parameters in the APACHE score led to a significant increase in disease severity score [5]. We acknowledge that both the SOFA and APACHE scores are not patient-centered outcome parameters. However, we have chosen these as primary outcomes over other parameters such as mortality(ratio) and length of hospital stay since these disease severity scores are calculated with data obtained close to the unplanned ICU transfer. Probably this data reflect the effect of monitoring at the general ward better compared to parameters that are more likely to be affected by factors during the ICU stay [23].

## Conclusion

The introduction of continuous vital sign monitoring on the hospital ward did not affect the disease severity of patients at the time of unplanned ICU transfer. This may suggest that continuous monitoring neither results in earlier nor delayed ICU transfer of patients who are clinically deterioration at the ward. The ICU length of stay, hospital length of stay, incidence of mechanical ventilation and ICU mortality were also not affected.

### Supplementary Information

Below is the link to the electronic supplementary material.

Supplementary file1 (DOCX 358 kb)

## Data Availability

On request (corresponding author).
