# Supplementary material for: The Effect of Continuous Versus Periodic Vital Sign Monitoring on Disease Severity of Patients with an Unplanned ICU Transfer
Source: J Med Syst. 2023 Mar 31;47(1):43. doi: 10.1007/s10916-023-01934-3 (PMC10066074; doi:10.1007/s10916-023-01934-3)
Supplement: Supplementary file 1 — Supplementary file1 (DOCX 358 kb) [file 10916_2023_1934_MOESM1_ESM.docx]

Appendix 1: Weighted contribution of vital parameters to MEWS and related protocol actions

|  | **Score points** | **3** | **2** | **1** | **0** | **1** | **2** | **3** |
| --- | --- | --- | --- | --- | --- | --- | --- | --- |
| A | Oxygen delivery, L/min |  |  |  | None | < 5 | ≥ 5 |  |
|  | Oxygen saturation, % | ≤ 91 | 92-93 | 94-95 | ≥ 96 |  |  |  |
| B | Respiratory rate, /min | ≤ 8 |  | 9-11 | 12-20 |  | 21-24 | ≥ 25 |
| C | Heart rate, /min | ≤ 40 |  | 41-50 | 51-90 | 91-110 | 111-130 | ≥ 131 |
|  | Systolic blood pressure, mmHg | ≤ 90 | 91-100 | 101-110 | 111-219 |  |  | ≥ 220 |
| D | Consciousness |  |  |  | A |  | Delirious | V/P/U |
| E | Core temperature, Celsius | ≤ 35.0 |  | 35.1-36.0 | 36.1-38.0 | 38.1-39.0 | ≥ 39.1 |  |

| **MEWS** | **Protocol action** |
| --- | --- |
| 0-2 | Next measurement <8 hours |
| 3-5 | Next measurement <4 hours, consultation fellow nurse or ward physician |
| ≥6 | Measurements every hour, consider continuous vital sign monitoring. Consultation ward physician within 10 min and/or Rapid Response Team (RRT) consultation |
|  | Be also aware of sepsis. |

AVPU-score: A; Alert (patient is awake and follows commands), V; Voice (patient does not respond to verbal stimulus), P; Pain (patient responds only to a painful stimulus), U; Unresponsive

Appendix 2: Nurses’ monitor at nurse station


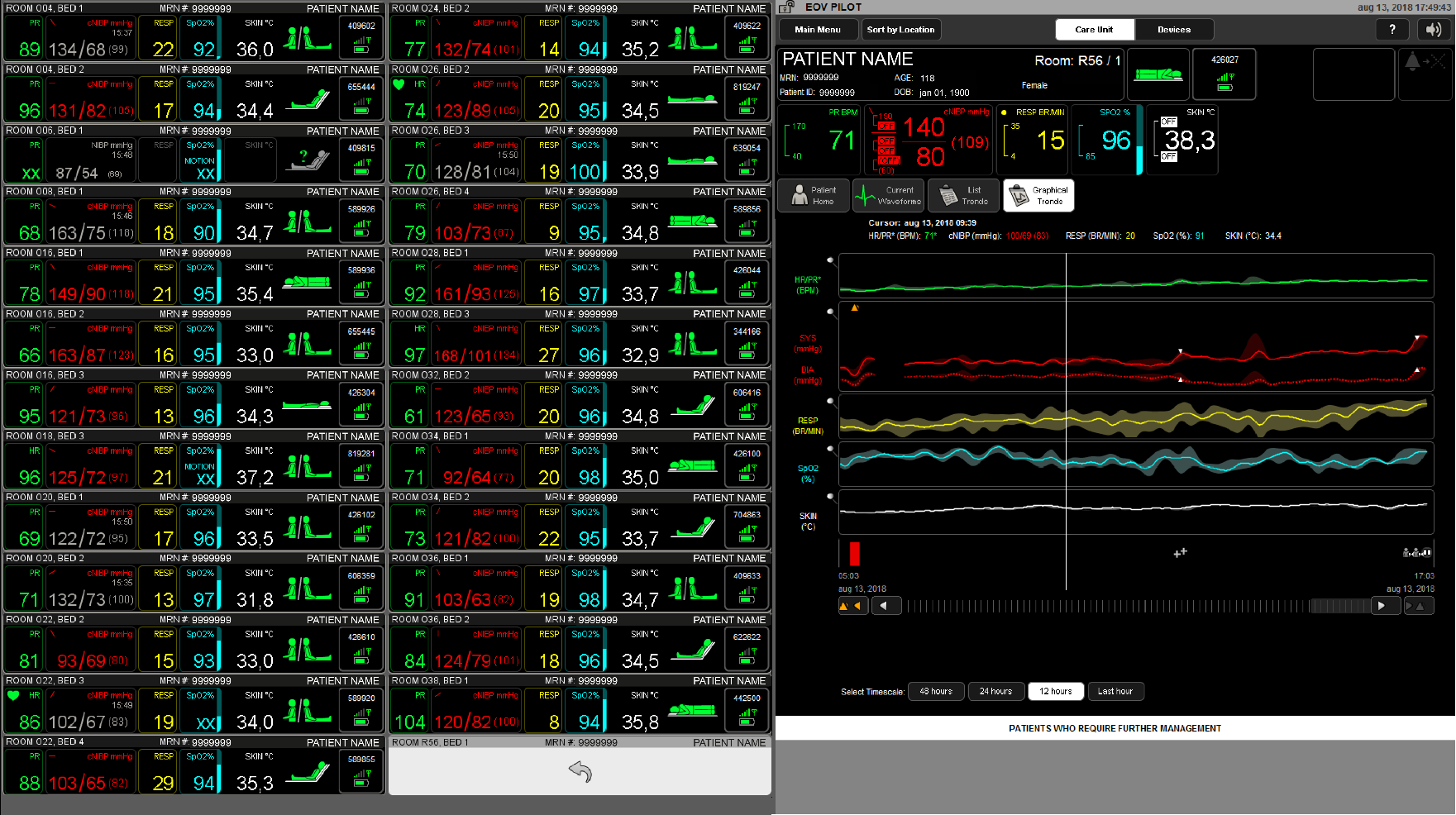


**Left half:** overview of the continuously monitored ward patients with the current vital signs, body position, device number, Wi-Fi signal strength and battery status shown. The white rectangle is a patient that is currently selected for a detailed view.

**Right half:** detailed view of selected patient. Patient details and current vital signs are shown at the top and the vital sign trends of the past 12 hours (possible up to 96 hours) are shown at the bottom half of the screen.

PR: pulse rate (heart rate based on oxygen saturation sensor; HR: heart rate (heart rate based on ECG); cNIBP: continuous non-invasive blood pressure; RESP: respiration rate; SpO_2_%: oxygen saturation; SKIN°C: skin temperature (not used by nurses, nor for this analysis). MRN: medical record number; DOB: date of birth; 🔍: button to open detailed view of individual vital sign trends.

Appendix 3: vital sign alarm setting continuous monitoring system

|  | Min | Max |
| --- | --- | --- |
| Oxygen Saturation (%) | 85 | - |
| Respiratory Rate (b/minute) | 4 | 35 |
| Heart Rate (b/minute) | 40 | 150 |
| Systolic blood pressure | - | 190 |
| Diastolic blood pressure | - | - |
| Mean arterial pressure (MAP) (mmHg) | 60 | - |
| Skin temperature (degrees Celsius) | - | - |
